# Supplementary material for: The causal relationship between the human gut microbiota and pyogenic arthritis: a Mendelian randomization study
Source: Front Cell Infect Microbiol. 2024 Nov 26;14:1452480. doi: 10.3389/fcimb.2024.1452480 (PMC11629706; doi:10.3389/fcimb.2024.1452480)
Supplement: Supplementary file 1 [file DataSheet1.zip › mendelian test/ebi-a-GCST90027604.csv_forest.pdf]

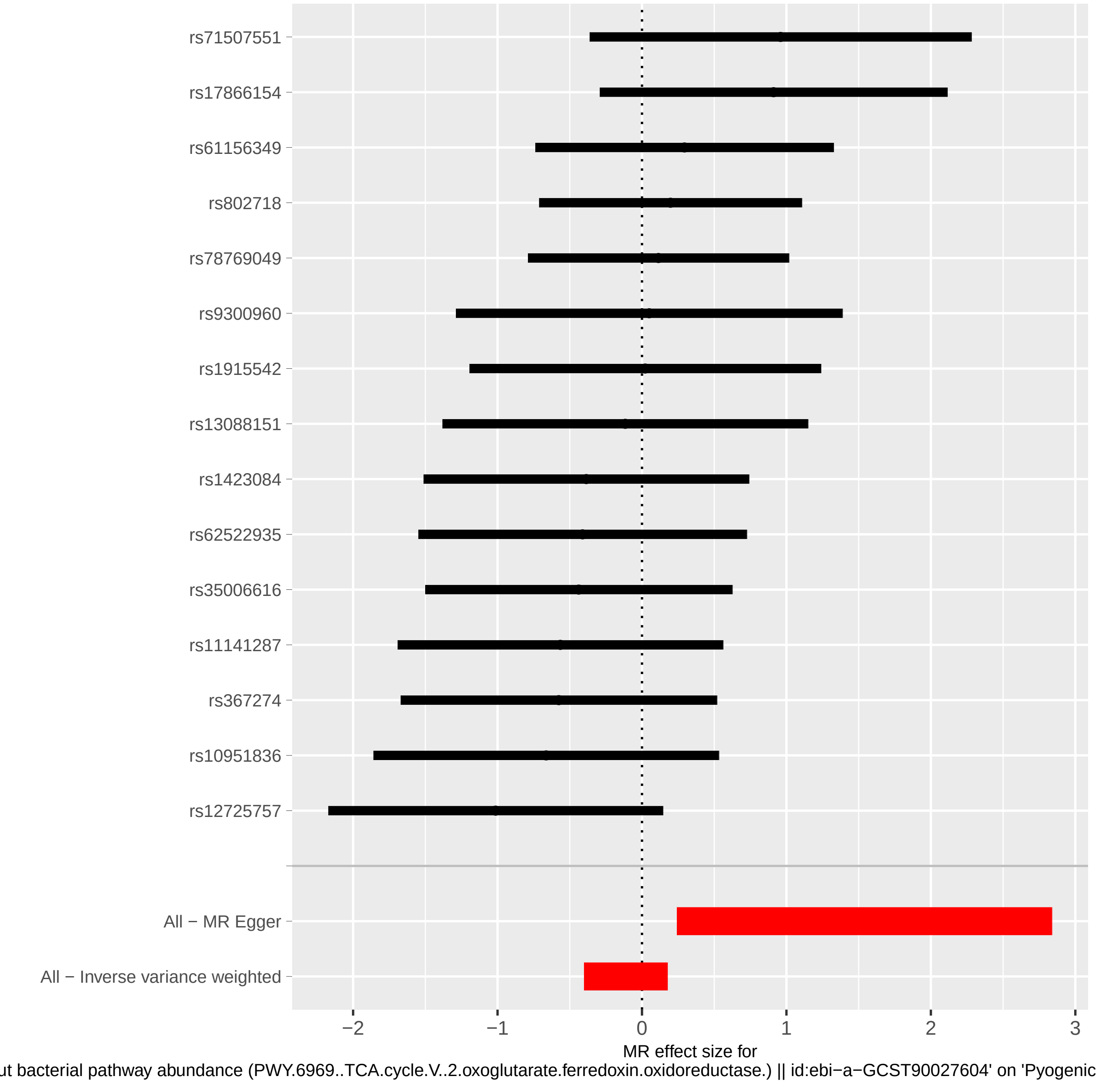

rs71507551
rs17866154
rs61156349
rs802718
rs78769049
rs9300960
rs1915542
rs13088151
rs1423084
rs62522935
rs35006616
rs11141287
rs367274
rs10951836
rs12725757
All − MR Egger
All − Inverse variance weighted
−2
−1
0
1
2
3
MR effect size for
ut bacterial pathway abundance (PWY.6969..TCA.cycle.V..2.oxoglutarate.ferredoxin.oxidoreductase.) || id:ebi−a−GCST90027604' on 'Pyogenic
